# Economic impact of switching to fixed-dose combination therapy for Japanese hypertensive patients: a retrospective cost analysis

**DOI:** 10.1186/1472-6963-13-124

**Published:** 2013-04-03

**Authors:** Manabu Akazawa, Katsushi Fukuoka

**Affiliations:** 1 Meiji Pharmaceutical University, Tokyo, Japan; 2 Nihon Chouzai Co., Ltd., Tokyo, Japan

**Keywords:** Antihypertensive drug, Fixed-dose combination, Economic benefit, Drug costs, Social experiment, Switching

## Abstract

**Background:**

The prescription of fixed-dose combinations (FDC) of antihypertensive drugs has increased rapidly since the relaxation of the prescription-term restriction.

In this study, we used the opportunity of this policy change in Japan as an instrument to assess the causal impact of switching to FDC on hypertensive treatment costs.

**Methods:**

Claims data from 64 community pharmacies located in Tokyo were used to identify hypertensive patients under continuous treatment with angiotensin-receptor blockers (ARBs). Patients switching to FDC between December 2010 and April 2011 were compared to patients who did not receive FDC (control group). Changes in annual antihypertensive drug costs were compared using a difference-in-differences approach to adjust for patient characteristics and use of concomitant medication. Subpopulation analyses were also performed, taking into account pre-index treatment patterns and prescribers’ characteristics.

**Results:**

There were 542 patients who switched to FDC and 9664 patients in the control group. No significant differences were observed between the 2 groups, except for antihypertensive drug use patterns before the policy change and prescribers’ characteristics. The switch to FDC was associated with an annual saving of 10,420 yen (US$112.0) in antihypertensive drug costs. Approximately 20% of the FDC patients, however, switched from ARB alone, and their drug costs increased by 2376 yen (US$25.5).

**Conclusions:**

For hypertensive patients who required ARB-based combination therapy, switching to FDC drugs had a significant cost-saving effect. However, the policy change of relaxing the prescription-term restriction could encourage aggressive treatment, i.e., switching to a combination therapy from monotherapy, regardless of medical conditions. Further research is required to evaluate the possible negative aspects of FDC drugs.

## Background

Aggressive antihypertensive treatment using a combination therapy that includes drugs with different mechanisms of action has been recommended as a means of achieving better blood pressure control [1-3]. Reflecting the clinical evidence, including the findings of the ACCOMPLISH study [4], an angiotensin-receptor blocker (ARB) together with a calcium-channel blocker (CCB) is the combination most frequently prescribed to Japanese hypertensive patients [5,6]. For patients who have comorbidities such as hyperlipidemia, diabetes, and chronic renal disease, more aggressive treatment, with the addition of thiazide diuretics (i.e., hydrochlorothiazide or HCTZ) is provided to achieve therapeutic goals [7]. Because those patients need to take multiple medications in a day, the complex regimen often affects the patients’ adherence to the treatments. Thus, fixed-dose combination (FDC) therapy becomes one option. Many studies suggested that simplifying drug regimens by reducing the number of pills may improve patient adherence, lower blood pressures, and save health service use and costs [8-15]. From the Canadian perspective, for example, a yearly estimated cost-saving of $27 to $45 million could be made when 60–100% of patients who had received 2 separate antihypertensive drugs switched to FDC products [16]. On the other hand, many antihypertensive drugs are available as generic formulations, while most FDC products are available as brand-name drugs alone. In the United States, where generics have the biggest market share in the world, observational studies using various databases indicated that the pharmacy cost of treating hypertension would increase after a switch to FDC products [11,14,17]. This increase cannot be ignored from the patients’ perspective, because their out-of-pocket costs vary according to their insurance status [18].

Since 2006, FDC pills that include ARB+HCTZ or ARB+CCB have been introduced to the Japanese market. By reflecting Japanese health policy to control drug expenditures by achieving a 30% market share in volume with generic drugs, the FDC pills were also expected to bring economic benefits [19,20]. Reimbursement prices (*Yakka*) of the FDC pills were set at 80% of the total prices of the individual drugs available in the market, where only branded ARBs were available [21]. The prices of the FDC antihypertensive drugs and their original drugs listed by the National Health Insurance (NHI) authority are summarized in Table 1. According to the list, by considering the cheapest combination, the daily price of ARB and HCTZ changes from 120.4 yen (valsartan + hydrochlorothiazide) to 120.9 yen (their FDC) and that of ARB and CCB changes from 137.9 yen (valsartan + amlodipine) to 120.3 yen (their FDC). However, since the market share of generic drugs (including HCTZ and CCB) is still low in Japan compared with other developed countries, while prescribers may reconsider their hypertensive treatment strategy when they prescribe an FDC, the impact on actual treatment costs of switching to the FDC drugs is uncertain and needs to be evaluated using real-world clinical data.

**Table 1:** NHI price list of antihypertensive drugs

	**Drug name**	**Listed price as of April 2012**	**Listed date of FDC drugs**
ARB+HCTZ FDC	losartan (50 mg) + hydrochlorothiazide (6.25 mg)	146.4	December 2006
	valsartan (80 mg) + hydrochlorothiazide (6.25 mg)	120.9	March 2009
	candesartan (8 mg) + hydrochlorothiazide (6.25 mg)	143.6	March 2009
	telmisartan (40 mg) + hydrochlorothiazide (6.25 mg)	137.9	June 2009
ARB+CCB FDC	valsartan (80 mg) + amlodipine (5 mg)	120.3	April 2010
	olmesartan (20 mg) + azelnidipine (16 mg)	158.1	April 2010
	candesartan (8 mg) + amlodipine (5 mg)	140.7	June 2010
	telmisartan (40 mg) + amlodipine (5 mg)	133.2	September 2010
ARB	losartan (50 mg)	143.4	
	valsartan (80 mg)	114.8	
	candesartan (8 mg)	140.4	
	telmisartan (40 mg)	131.0	
	olmesartan (20 mg)	130.4	
HCTZ	hydrochlorothiazide (25 mg)	5.6	
CCB	amlodipine (5 mg)	23.1	
	azelnidipine (16 mg)	65.5	

*ARB*: angiotensin-receptor blocker, *CCB*: calcium-channel blocker, *FDC*: fixed-dose combination, *HCTZ*: hydrochlorothiazide.

This table created from the NHI Drug Price List as of April 2012. When various doses of combination drugs exist, a major combination that is used frequently was selected. A generic version of losartan was listed in June 2012 at a price of 86.0 yen for a 50 mg tablet, about 60% of the cost of the original drug.

In Japan, newly listed drugs can be prescribed only in quantities sufficient for 14 days’ treatment [22]; therefore, patients have to visit their doctors repeatedly to get their prescriptions. This dispensing-day restriction is removed 1 year after launch, except for narcotic and psychotropic drugs. In a survey of 490 Japanese doctors, over 80% responded that the 14-day dispensing rule influences their choice of treatment [23]. Considering their patients’ inconvenience, doctors often hesitate to prescribe new medications if there are other options, especially when they are treating conditions that require continuous management, such as hypertension. Thus, the market penetration of new drugs can be somewhat limited by this restriction. In the case of FDC drugs, however, Japan’s Central Social Insurance Medical Council (*Chuikyo*) decided that, because the clinical experience of each individual drug was sufficient, the14-day dispensing rule could be relaxed [24] and long-term prescriptions of all FDC antihypertensive drugs, even those newly listed, have been allowed since December 10, 2010. This policy change appeared likely to influence doctors’ prescribing behavior, so that the number of prescriptions of FDC drugs would increase rapidly immediately after this restriction was removed. In some cases, doctors might switch antihypertensive drugs regardless of medical conditions that required more aggressive treatments to control blood pressure.

In many previous studies that evaluated the benefits of FDC drugs, annual medical and drug costs were compared in patients who used FDC drugs and those without FDC drugs. To minimize potential selection bias, patient background characteristics were adjusted using propensity score and/or multivariable regression techniques [9,13,15,17]. Because limited information is available in healthcare databases, unobservable confounding cannot be eliminated. Therefore, using this policy change in Japan as an instrument that would be endogenous for doctor preference and patient case-mix, we tested the hypothesis that switching to FDC drugs actually reduces antihypertensive drug costs, using a real world prescription record in Japan.

## Methods

### Data source

Nihon Chouzai is a large community pharmacy chain that has 457 dispensing pharmacies throughout Japan. We used its pharmacy claims database to extract data for patients who were prescribed ARBs continuously (i.e., who had dispensing records of an ARB at least every 4 months between December 2009 and March 2012) from pharmacies located in the Tokyo area (64 branches). The information stored in the database includes anonymous identification codes of patients, pharmacies, and institutions; patients’ age and gender; dispensing date; drug name (brand and general names, and unique identification code, called the “YJ code”); and drug price, dose, and duration (dispensing days).

### Study population and design

**Figure 1:**
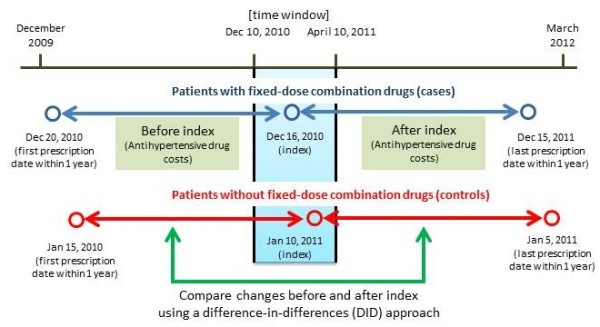
**Study design.** Cases were defined as patients who started taking fixed-dose combination (FDC) drugs within the time window. The index date was defined as the date of first prescription within the time window of FDC for cases and of ARB for controls. The total and antihypertensive drug costs were calculated before and after the index date and were compared in cases and controls. A difference-in-differences (DID) approach was used to estimate the effect on annual costs of switching to FDC drugs.

The relaxation of the 14-day dispensing rule for newly listed FDC drugs started on December 10, 2010. Therefore, on the assumption that hypertensive patients visit the pharmacy at least once every 4 months for their continuous treatment, a 4-month time window (from December 10, 2010 to April 10, 2011) was set to capture patients who switched antihypertensive drugs in response to the policy change (Figure 1). We identified patients who started taking fixed-dose ARB+HCTZ or ARB+CCB drugs during this time window; these were designated as cases. Patients who did not have dispensing records of FDC drugs but were taking an ARB were assigned to the control group. For both cases and controls, the first date within the time window on which the FDC drug or ARB was dispensed was set as the index date. Patients who started FDC therapy before or after the time window or who did not have any record of ARB dispensing were excluded from the study population.

### Variables

The main outcomes of interests were the annual antihypertensive drug costs before and after the index date. The drug costs were summed over the 1-year interval prior to the index date (as pre-index costs) and over the 1-year interval after the index date (as post-index costs), including all prescriptions dispensed within the respective intervals. The annual costs were then calculated by adjusting for differences between 365 days and the actual follow-up days from which the costs were derived. Antihypertensive drugs included were ARBs, angiotensin-converting enzyme (ACE) inhibitors, dihydropyridine CCBs, thiazide diuretics (HCTZ), beta-blockers, alpha-beta blockers, and the FDC drugs. The study design is illustrated in Figure 1, which shows an example of how the timeframe was defined. Information about gender, age categories (≤64 years, 65–74 years, and ≥75 years), the number of drugs taken (use of ≥7 drugs), use of a diabetes drug, and use of a hyperlipidemia drug was extracted from the prescription records during the 4-month time window to create variables that would represent the patients’ background characteristics. As for prescribers’ characteristics, institutions where the prescriptions at the index date were issued were classified into 2 and variables indicating clinic (no bed) or hospital, as well as cardiovascular specialists or others, were created.

### Analysis

The prescription pattern of the FDC drugs was summarized as the ratio of the FDC prescriptions per month to the total ARB prescriptions (including FDC drugs) per month. A comparison of the patient characteristics (χ2 test) and annual antihypertensive drug costs (*t*-test) was made between the FDC drug users and the controls. Among the FDC drug users, the patients who switched from ARBs alone, those who had been taking both ARB and CCB in separate forms, those whose drugs were prescribed by doctors at clinics, and those whose drugs were prescribed by cardiovascular specialists, were selected as subgroups to compare the changes in antihypertensive drug costs. Because the dispensing fees paid to doctors by the NHI are based on the number of drugs prescribed, we evaluated the association between taking ≥7 drugs and switching to FDC drugs. Dispensing fees are discounted when ≥7 drugs are prescribed at the same time, as a disincentive to polypharmacy [25].

Changes in the drug costs among the FDC drug users were compared with changes among the non-FDC drug users (controls) using a difference-in-differences (DID) approach [26]. The effect of the policy change (switching to FDC drugs) on drug costs (δ) was estimated by the following equation.

$$\begin{array}{cc} \;\delta = & \left({\mathrm{COST}}_{\mathrm{post}-\mathrm{index}\_\mathrm{case}}-{\mathrm{COST}}_{\mathrm{pre}-\mathrm{index}\_\mathrm{case}}\right)\\ & -\left({\mathrm{COST}}_{\mathrm{post}-\mathrm{index}\_\mathrm{control}}-{\mathrm{COST}}_{\mathrm{pre}-\mathrm{index}\_\mathrm{control}}\right) \end{array}$$

Estimated costs were expressed in yen (1 US$ = 93 yen as of February 14, 2013).

The impact of patient characteristics, including gender, age categories, number of concomitant drugs taken, diabetes treatment, and hyperlipidemia treatment, was adjusted using a multivariable regression method. The level of statistical significance was set at 5%. All statistical analyses were performed using SAS software version 9.3 (SAS Institute Inc., 2012, Cary NC USA). The study was approved by the Institutional Review Board at Meiji Pharmaceutical University (study number 2305) and was conducted in compliance with the Japanese Ethical Guidelines for Epidemiological Research, as updated in December 2008 [27].

## Results

**Figure 2:**
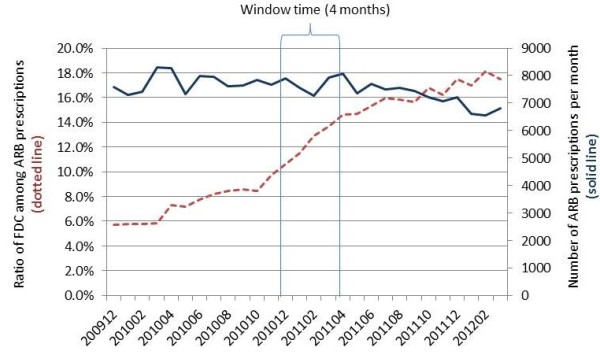
**Changes in prescription pattern during the study period.** The dotted line indicates the ratio of FDC drugs to the total ARB prescriptions and the solid line indicates the number of ARB prescriptions (including FDC drugs) per month. A 4-month time window from December 2010 to April 2011 was set to capture FDC switching after the policy change relaxing the prescription-term restriction.

From the database we identified 11,993 hypertensive patients who received ARB prescriptions regularly for 2 years from 64 pharmacies located in Tokyo. The number of prescriptions for ARBs (including FDC drugs) was fairly constant, varying mainly between 7000 and 8000 per month, during the follow-up period (Figure 2). During that period, the ratio of FDC prescriptions to total ARB prescriptions increased rapidly, especially during the time window, from 10% in December 2010 to 15% in April 2011.

**Figure 3:**
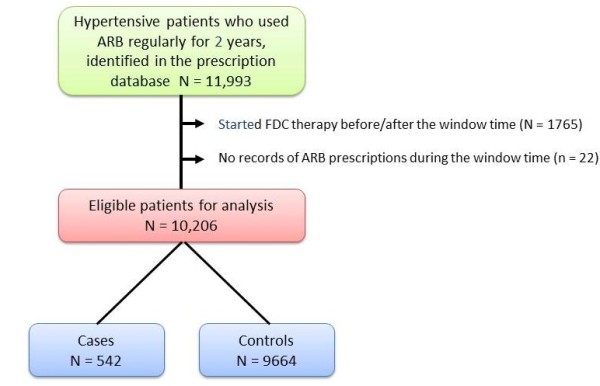
**Flowchart showing the selection of the study population.** ARB: angiotensin-receptor blocker, FDC: fixed-dose combination. Index date was defined as the first prescription date of FDC or ARB during the window time.

To evaluate the impact on drug costs of switching to FDC drugs, patients who started FDC therapy before and after the time window (n = 1765) and those who had no ARB prescription records during the time window (n = 22) were excluded (Figure 3). The eligible study population was then 10,206, comprising 542 cases who switched to FDC drugs and 9664 controls who did not. Among the 542 cases, 243 received fixed-dose ARB+HCTZ and 302 received fixed-dose ARB+CCB, including duplicated cases.

**Figure 4:**
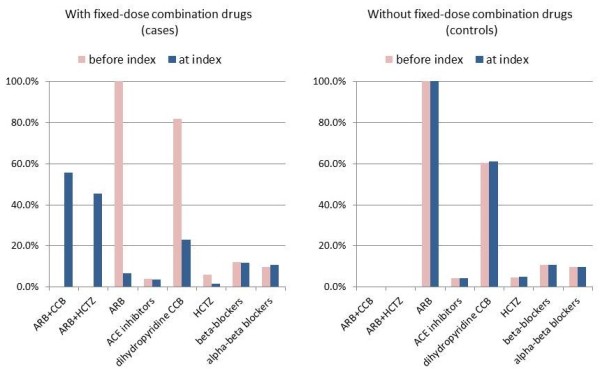
**Prescription patterns of antihypertensive drugs among cases and controls.** ACE: angiotensin-converting enzyme, ARB: angiotensin-receptor blocker, CCB: calcium-channel blocker, HCTZ: hydrochlorothiazide

The characteristics of the cases and controls are summarized in Table 2. Gender and age were similarly distributed in the 2 groups. Cases were more likely to have been taking ARB and CCB combination therapy before the index date (74.7% vs. 54.5%) and controls were more likely to use ARB alone (40.7% vs. 19.2%). More switches occurred when prescriptions were issued by doctors at clinics (20.5% vs. 10.3%) and by cardiovascular specialists (31.7% vs. 25.4%). No differences were observed in the numbers of concomitant drugs, or in the use of drugs for diabetes or hyperlipidemia. According to the prescription pattern illustrated in Figure 4, the majority of hypertensive patients were treated with ARBs and dihydropyridine CCBs, as expected. However, for the cases, the proportion of these drugs was dramatically reduced after the index date as they were replaced by the FDC drugs.

**Table 2:** Patient characteristics

	**With fixed-dose combination drugs (cases)**		**Without fixed-dose combination drugs (controls)**		**p (χ2 tests)**
Number of patients	542		9664		
Gender					0.1735
Male	198	36.5%	3814	39.5%	
Age category					0.2087
≤64 years	212	39.1%	3729	38.6%	
65–74 years	153	28.2%	3047	31.5%	
≥75	177	32.7%	2888	29.9%	
Drug use before index					<.0001
ARB and CCB combination	405	74.7%	5269	54.5%	
ARB alone	104	19.2%	3938	40.7%	
Number of concomitant drugs					0.4567
7 or more	74	13.7%	1214	12.6%	
Diabetes drugs					0.0582
Users	153	28.2%	2379	24.6%	
Hyperlipidemia drugs					0.8094
Users	236	43.5%	4157	43.0%	
Prescribers’ characteristics					
Doctors at clinics (no bed)	111	20.5%	993	10.3%	<.0001
Cardiovascular specialists	172	31.7%	2457	25.4%	0.0011

*ARB*: angiotensin-receptor blocker, *CCB*: calcium-channel blocker.

**Figure 5:**
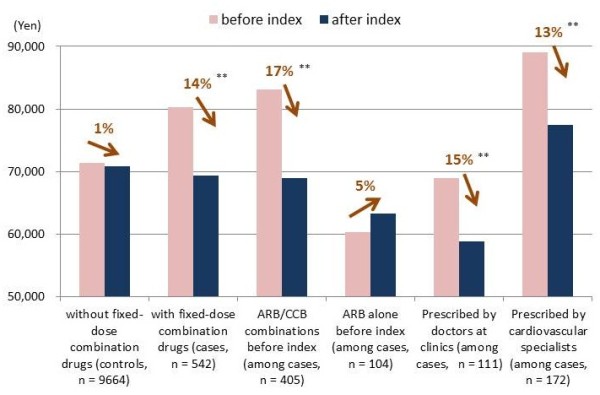
**Antihypertensive drug costs before and after index date for various patient populations.** ARB: angiotensin-receptor blocker, CCB: calcium-channel blocker. Changes in antihypertensive drug costs were statistically significant at 1% (**) levels according to *t*-tests.

Annual drug costs before and after the index date are summarized in Figure 5. Among controls, drug costs slightly decreased (by 1% or 589 yen) after the index date, while large savings were observed (by 14% or 10,999 yen) for patients who started taking FDC drugs (cases). In order to investigate these changes further, we selected subpopulations according to the pre-index treatment patterns—patients treated with combined ARB and CCB in separate forms (n = 405) and those treated with ARB alone (n = 104) —as well as prescribers’ characteristics—doctors at clinics (n = 111) and cardiovascular specialists (n = 172). Statistically significant cost-savings were observed for patients switching from the combination therapy of ARB and CCB (by 17% or 14,079 yen), prescribed by doctors at clinics (by 15% or 10,132 yen), and by specialists (by 13% or 11,684 yen). On the other hand, the annual drug costs were increased for patients switching from ARB alone to FDC therapy (by 5% or 2965 yen).

The changes in the costs were estimated using the DID approach by adjusting for patient characteristics and concomitant medications (Table 3). For the study population as a whole, cost-saving effects were observed (10,420 yen on average). In addition, statistically significant cost reductions were observed for the cases who switched from the combination of ARB and CCB (12,800 yen on average), prescribed by doctors at clinics (8815 yen on average), and by cardiovascular specialists (11,081 yen on average). However, for those who switched from ARB alone, a negative impact on costs (2376 yen increase on average) was observed, though it was not statistically significant.

**Table 3:** Results of DID estimations of antihypertensive drug costs

	**Estimate**	**Standard error**	**t**	**p**
Total patients n = 10,206	-10420	672	-15.51	<.0001
ARB and CCB combination before index n = 5674	-12800	836	-15.32	<.0001
ARB alone before index n = 4042	2376	1252	1.90	0.0577
Prescribed by doctors at clinics n = 1104	-8815	1411	-6.25	<.0001
Prescribed by cardiovascular specialists n = 2629	-11081	1302	-8.51	<.0001

Difference-in-differences (DID) estimates were obtained by adjusting for gender, age category, number of drugs (≥7), diabetes drug users, and hyperlipidemia drug users. Antihypertensive drug costs were calculated by sum of reimbursement costs for angiotensin-receptor blockers (ARB), angiotensin-converting enzyme inhibitors, dihydropyridine calcium-channel blockers (CCB), thiazide diuretics, beta-blockers, alpha and beta-blockers, and fixed-dose combination drugs.

## Discussion

This study provides evidence that switching to FCD drugs reduces the annual cost of antihypertensive treatments. In particular, for patients treated with a combination of ARB and CCB in separate forms, the estimated annual savings are of the order of 12,800 yen. On the other hand, one-fifth of the patients switched from a single ARB pill to the FDC drug during the time window. This suggests that relaxing the 14-day dispensing rule may trigger more aggressive treatment to achieve better blood pressure control and could increase annual drug costs in such cases by 2400 yen.

A number of studies have indicated that switching to FDC drugs improves medication adherence [10,28-30]. However, we could not confirm this benefit using pharmacy claims data in Japan. Because patients visited the doctor’s office regularly and received medications according to a schedule, the medication possession ratio (MPR) —which is defined as the total days of supply of drugs during the study period divided by the length of the follow-up period and is often used to measure adherence in claims-based studies [31] —was almost 100% for most patients, regardless of whether they actually took the medications as indicated or not. In April 2012, the NHI reimbursement rule for dispensing fees was revised in order to increase the pharmacists’ responsibility for checking for unused drugs and adjusting the quantity of drugs dispensed [25,32]. When patients receive medications only as necessary, the MPR method can be used to monitor their medication adherence using the claims.

We assumed that doctors might have an incentive to use the FDC drugs so as to reduce the number of medications. According to Japan’s NHI reimbursement rule, doctor’s prescription fees are 400 yen for prescribing ≥7 drugs compared with 680 yen for prescribing ≤6 drugs. We identified 12% of the study population who had a prescription for ≥7 drugs. A sub-analysis targeting only those patients (data not shown) showed that the proportion who switched to FDC drugs and the impact on drug costs were almost the same as in the total study population. This finding suggests that doctors do not care about prescription fees; therefore, the switch may occur regardless of the number of medications that are prescribed at the same time.

The change in the dispensing rule encouraged the use of an FDC drug. Due to the NHI pricing rule for ARB combination drugs (approximately 80% of the individual drugs), for most patients the switch resulted in a cost saving. However, some cases switched from treatment with ARB alone, resulting in an increased cost. If these prescription changes occurred due to promotion of the new drugs, rather than to medical need, the economic benefits of switching need to be examined with care. It should also be noted that a generic version of the ARB losartan was introduced in June 2012 at prices 45% below those of the corresponding branded medication (45.3 yen for 20 mg, 86.0 yen for 50 mg, and 129.0 yen for 100 mg). Under Japanese dispensing rules, generics can be dispensed by pharmacists without the doctor’s permission, unless the prescription form specifies that there should be no generic substitution [33]. However, once a patient has switched to an FDC, a further change to generics is unlikely, because the pharmacist would need to ask the doctor to rewrite the prescription to show the ARB and other medication separately before dispensing the generic substitutions—a rather unrealistic scenario. Thus, as our estimates suggest that the maximum cost saving from switching to FDC drugs was 17%, greater savings can be expected if patients continue to use separate ARB and CCB medications and switch to the generic forms when they become available. These effects need to be monitored constantly using pharmacy claims.

This social experiment examined the economic impact of switching to FDC antihypertensive treatment in terms of the benefit to the patient. However, because some patients actually switched to the aggressive treatment because of their medical needs, the effects we observed could be biased toward either direction. In addition, we had access only to prescription records at community pharmacies, whereas no clinical information, such as diagnosis and blood pressure, was available. Thus, the results should be adjusted for lifestyle diseases that might influence the treatment costs, incorporating variables corresponding to the use of drugs for diabetes and hyperlipidemia. Furthermore, patients may visit different pharmacies to obtain their medications and this would not have been apparent from the available pharmacy data. However, because the MPR was calculated as 100% for many patients, these misclassifications might be negligible. The DID approach was used to evaluate the effect of policy changes using claims data [34]. In addition, we could adopt a time-series approach, using monthly records of claims to compensate for the inherent limitation of unmeasured variables, especially targeting cases who switched from ARB alone to the FDC drugs [35].

## Conclusions

There are economic benefits from using combination pills when the switch occurs from combination therapy with the same drugs in separate forms. However, the introduction of the combination pills may result in aggressive but unnecessary treatment. Further research is needed to evaluate the economic impact of the combination pills by considering the introduction of antihypertensive generics in the future market.

## Abbreviations

ACE: Angiotensin-converting enzyme; ARB: Angiotensin-receptor blocker; CCB: Calcium-channel blocker; DID: Difference-in-differences; FDC: Fixed-dose combination; HCTZ: Hydrochlorothiazide; MPR: Medication possession ratio; NHI: National health insurance

## Competing interests

The authors declare that they have no competing interests.

## Authors’ contributions

MA conceived the study. All authors contributed to the study design. KF was responsible for extracting the pharmacy claims data. MA was responsible for the data analysis and drafting the manuscript. All authors read and approved the final version of the manuscript.

## Pre-publication history

The pre-publication history for this paper can be accessed here:

http://www.biomedcentral.com/1472-6963/13/124/prepub
